# Institutional Notes and News

**Published:** 1911-08-19

**Authors:** 


### Institutional Notes and News.

**New Pay-Hospital at Leicester.**

A Correspondent in Leicester informs us that: "Earl Howe has opened the new hospital which has been built at Leicester in connection with the Leicester and Leicestershire Provident Dispensary. There has been a need in Leicester for a hospital for those who, not able to provide for treatment in private nursing homes and not wishing to obtain free treatment in the Leicester Infirmary, could be provided for on payment of moderate fees. For some time the Dispensary Committee had provided five beds for such patients, but the increasing demand for accommodation necessitated a larger and more permanent structure. The new hospital contains three wards, 39 feet in length and 24 feet in width, each accommodating ten beds. The operating theatre is on the central floor. Throughout the building the walls are of parian cement, painted and varnished, and the ward floors of teak. The entrance hall, landings, operating theatre, and sanitary annexes are floored with terrazzo-marble mosaic. The administration block contains a waiting-room, matron's room, nurses' sitting-room, servants' room, scullery, kitchen, and bathrooms. The heating system and the mortuary are in the basement. The cost of the hospital is estimated at £4,000, and the architects are Messrs. Stockdale, Harrison, and Son, and the builders Messrs. Herbert, both of Leicester. Mr. John E. Faire, J.P. (Chairman of the Dispensary), presided over the opening ceremony, and announced that Sir Edward Wood, J.P., the Chairman of the Infirmary, generously had sent £500 to the building fund, to which a sum of £1,500 had been contributed."

---

**Hospital Work in Yorkshire.**

A Yorkshire correspondent writes: Probably in no part of the country is such activity in the direction of carrying out hospital improvements being displayed as that within a small, yet densely crowded, area in the West Riding of Yorkshire. This area covers the woollen district, and embraces the cities of Leeds and Bradford and the townships of Batley and Dewsbury—all these places being in near contiguity one to the other. At Leeds, as has previously been reported on many occasions in The Hospital, efforts are being made towards raising the sum of £150,000 for works of reconstruction and improvement; at Bradford the erection of a new infirmary on modern lines has advanced a step further by the acceptance of designs for the building; the people of Dewsbury are taking a deep interest in the local King Edward Memorial proposals; and at Batley the matter of hospital extensions last week formed the subject of a Local Government Board inquiry in the heavy woollen town. The scheme in the last-mentioned town is that of the Oakwell Joint Hospital Board, and it is to increase the accommodation so that instead of there being only twenty-six beds (as at present) there will be sixty beds. The application was made by the Oakwell board, and the increased accommodation, it was pointed out, was necessitated by the recent inclusion of Batley in the area of the board. Sanction was sought to borrow the sum of £4,000, and when the proposed extensions are carried out it was stated that the total cost of the hospital would amount to close upon £20,000. The application for borrowing powers received strong support, and there was no opposition.

---

**Cambridge Memorial to King Edward.**

At a meeting convened by his Worship the Mayor of Cambridge, and presided over by the Lord Lieutenant of the county, Viscount Clifden, at the Guildhall, Cambridge, last autumn (The Hospital of November 5, 1910, page 181), it was resolved that the memorial should take the form of a fund to provide Addenbrooke's Hospital with the much-needed new wards for children and a new out-patient department, as well as several other improvements to fit it for the growing needs of the very large district which it serves—namely, the whole of the County of Cambridge and portions of the Counties of Herts, Hunts, Norfolk, Suffolk, Essex, Bedford, Northampton, and Lincoln—particularly with regard to the accommodation required for children and out-patients. The Fund was closed on June 20 last, and amounted to £10,595, which includes special donations amounting to £112 12s. for a statue, bust, or picture. To this must be added bank interest £106 3s. 9d., bringing the total of the Fund up to £10,702. This contribution will be a great help towards the sum of some £18,000 or £20,000 which is required to provide the new wards for children, new out-patient department, new steam boilers and boiler house, additional accommodation for nurses, electric lift in main building, etc. If a statue is to be erected a considerable addition to the £112 12s. already subscribed will be necessary. In this connection it is important to add that the Committee of the Hospital will be pleased to receive further contributions from old Cambridge students, with a view if possible to increase still further the above-mentioned sum handed over by the Memorial Fund Committee to £18,000 or £20,000. The provision of the new wards for children and new out-patients' department, it is estimated, will increase the annual expenditure by about £1,000 per annum. Towards this the Committee will welcome the annual contribution from the Cambridge and District Workers' Hospital Fund, which it is believed will amount to at least £400 for the first year, and will steadily increase every twelve months.

